# Prevalence and genetic diversity of avian haemosporidian parasites in wild bird species of the order Columbiformes

**DOI:** 10.1007/s00436-021-07053-7

**Published:** 2021-02-01

**Authors:** Yvonne R. Schumm, Dimitris Bakaloudis, Christos Barboutis, Jacopo G. Cecere, Cyril Eraud, Dominik Fischer, Jens Hering, Klaus Hillerich, Hervé Lormée, Viktoria Mader, Juan F. Masello, Benjamin Metzger, Gregorio Rocha, Fernando Spina, Petra Quillfeldt

**Affiliations:** 1 Department of Animal Ecology & Systematics, Justus Liebig University, Heinrich-Buff-Ring 26-32, 35392 Giessen, Germany; 2 Aristotle University of Thessaloniki, School of Forestry and Natural Environment, Lab. of Wildlife & Freshwater Fish, PO Box 241, University Campus, 54124 Thessaloniki, Greece; 3 Antikythira Bird Observatory, BirdLife Greece, Athens, Greece; 4 Area Avifauna Migratrice, Istituto Superiore per la Protezione e la Ricerca Ambientale (ISPRA), Via Ca’ Fornacetta 9, I-40064 Ozzano dell’Emilia, Italy; 5 OFB–Unité Avifaune Migratrice, Direction de la Recherche et de l’appui Scientifique, Carrefour de la Canauderie, 79360 Villiers en Bois, France; 6 Clinic for Birds, Reptiles, Amphibians and Fish, Veterinary Faculty, Justus Liebig University Giessen, Frankfurter Strasse 114, Giessen, Germany; 7 Verein Sächsischer Ornithologen e.V., Wolkenburger Straße 11, 09212 Limbach-Oberfrohna, Germany; 8 Röntgenstraße 7, 64823 Groß-Umstadt, Germany; 9 26/1 Immaculate Conception Street, Gzira, GZR 1141 Malta; 10 Department of Zoology, Veterinary School, University of Extremadura, Avda de las Ciencias s/n, 10003 Cáceres, Spain

**Keywords:** Avian malaria, Woodpigeon, Turtle dove, Stock dove, Parasite ecology

## Abstract

Diseases can play a role in species decline. Among them, haemosporidian parasites, vector-transmitted protozoan parasites, are known to constitute a risk for different avian species. However, the magnitude of haemosporidian infection in wild columbiform birds, including strongly decreasing European turtle doves, is largely unknown. We examined the prevalence and diversity of haemosporidian parasites *Plasmodium*, *Leucocytozoon* and subgenera *Haemoproteus* and *Parahaemoproteus* in six species of the order Columbiformes during breeding season and migration by applying nested PCR, one-step multiplex PCR assay and microscopy. We detected infections in 109 of the 259 screened individuals (42%), including 15 distinct haemosporidian mitochondrial cytochrome *b* lineages, representing five *H*. (*Haemoproteus*), two *H*. (*Parahaemoproteus*), five *Leucocytozoon* and three *Plasmodium* lineages. Five of these lineages have never been described before. We discriminated between single and mixed infections and determined host species-specific prevalence for each parasite genus. Observed differences among sampled host species are discussed with reference to behavioural characteristics, including nesting and migration strategy. Our results support previous suggestions that migratory birds have a higher prevalence and diversity of blood parasites than resident or short-distance migratory species. A phylogenetic reconstruction provided evidence for *H*. (*Haemoproteus*) as well as *H*. (*Parahaemoproteus*) infections in columbiform birds. Based on microscopic examination, we quantified parasitemia, indicating the probability of negative effects on the host. This study provides a large-scale baseline description of haemosporidian infections of wild birds belonging to the order Columbiformes sampled in the northern hemisphere. The results enable the monitoring of future changes in parasite transmission areas, distribution and diversity associated with global change, posing a potential risk for declining avian species as the European turtle dove.

**Supplementary Information:**

The online version contains supplementary material available at 10.1007/s00436-021-07053-7.

## Introduction

There is increasing evidence that pathogens can play a significant role in species decline (Bunbury et al. 2007). Haemosporidian parasites, including *Plasmodium*, known as avian malaria, and related malaria-like pathogens *Leucocytozoon* and subgenera *Haemoproteus* and *Parahaemoproteus* have been associated to negatively affect bird population dynamics (Yanga et al. 2011; Yoshimura et al. 2014). Several studies demonstrated different costs on life-history traits associated with haemosporidian infections, such as impairment on the body condition (Valkiūnas et al. 2006), reduced reproductive success (Merino et al. 2000; Marzal et al. 2005; Knowles et al. 2010) and lower chance of survival (Earle et al. 1993; Sol et al. 2003; Bunbury et al. 2007; Lachish et al. 2011).

Haemosporidian parasites are widespread and infect a great variety of avian host species (Valkiūnas 2005; Boundenga et al. 2017). Nevertheless, most studies have specifically addressed avian haemosporidians of passerine birds, while research on non-passerine host species is underrepresented (Santiago-Alarcon et al. 2010; Clark et al. 2014). There is only a small number of recent publications dealing with haemosporidian parasites in wild columbiform birds, particularly in Europe, apart from feral pigeon *Columba livia domestica* (e.g. Sol et al. 2003; Foronda et al. 2004; Scaglione et al. 2015).

In general, given their common evolutionary background, closely related host species (i.e. species belonging to the same family) are expected to be similar in their susceptibility to parasitic infestations and exposure to vectoring dipterans and their parasite community (Ricklefs and Fallon 2002; Dubiec et al. 2016; Ciloglu et al. 2020a; Ellis et al. 2020). However, only few studies have presented data on the prevalence and diversity of haemosporidian parasites in closely related bird species. Differences in prevalence between species are associated with several factors and the interactions between those, including life-history traits and ecology of the hosts and vectors, parasite characteristics and environmental conditions, that may affect the activity of vectors and the development of parasites (Sol et al. 2000; Gupta et al. 2011; Quillfeldt et al. 2011; Hellard et al. 2016; Chakarov et al. 2020; Ciloglu et al. 2020b; Ellis et al. 2020). Also different behavioural characteristics (e.g. cavity-nesting vs. open-nesting or migrant vs. resident species) may influence haemosporidian prevalence and community (Dunn et al. 2017; Emmenegger et al. 2018), whereas no evidence that closely related host species share parasites due to overlapping geographic ranges was found (Ciloglu et al. 2020a). Cavity-nesting species may be shielded from vector exposure due to their enclosed surroundings, while open-nesting birds should be more susceptible to flying dipteran vectors. Migratory species, particularly long-distance migrants, are expected to host a higher diversity of parasites (Walther et al. 2016; Emmenegger et al. 2018; Ciloglu et al. 2020b) as they encounter parasites and their vectors in multiple ecosystems each year, whereas residents only encounter parasites in one ecosystem (Møller and Erriyzøe 1998). The European turtle dove *Streptopelia turtur* (henceforth turtle dove) is the only long-distance migrant among the columbiform birds we tested. The European population follows three main migration flyways (western, central and eastern) between Europe and sub-Saharan Africa (Marx et al. 2016). The population trend of turtle doves across Europe declined by almost 80% since the 1970s, whereas population trends of other columbiform species, like Common woodpigeon *C. palumbus* (henceforth woodpigeon) and stock dove *C. oenas*, are increasing (PECBMS 2020). Stock doves and woodpigeons from Central Europe are partial migrants. Migratory individuals are mainly wintering in France and Iberia (Cramp 1985; von Blotzheim and Bauer 1994). The main reasons for the turtle dove population decline are the loss of good-quality habitats as well as illegal and unsustainable legal hunting. Additional threats were identified, but these are either considered to have a small or unknown impact or need further research (Fisher et al. 2018); among these are diseases like haemosporidian infections.

We used molecular and microscopic techniques to screen the columbiform species for haemosporidian infections and to identify genetic lineages in order to test the following hypotheses: (i) the prevalence of haemosporidian parasites is higher in long-distance compared to short-distance migratory or resident species, (ii) the diversity of lineages differs among related species and (iii) the prevalence and lineage occurrence in turtle doves varies across their flyways due to possible differing parasite-vector-communities at different breeding, stopover and wintering areas.

## Material and methods

### Origin and preparation of the samples

Blood samples from 259 individuals belonging to six species of the order Columbiformes were collected from 2013 to 2019 over a broad geographical extent (Table 1; Fig. S1) by venipuncture of the brachial or metatarsal vein and stored on Whatman FTA cards (Whatman®, UK). A blood smear was prepared in the field for 251 of the sampled birds. The blood smears were fixed with methanol (100%) for 30 s and stained with Giemsa in a work solution prepared with buffer pH 7.0 (ratio 1:5) for 30 min. For DNA isolation, a 3 × 3 mm piece of each sample was cut out of the FTA card. Subsequently, the DNA was extracted according to the ammonium-acetate protocol by Martínez et al. (2009) and purified with Zymo-SpinTM IIC columns (Zymo Research, USA). DNA concentration and purity were quantified by using NanoDrop2000c UV-Vis spectrophotometer (NanoDrop Technologies, USA).

**Table 1:** Number of blood samples analysed, split by species year and site

Country	Location	Species^a^	Sampling year	Sampling period^b^	Sample size FTA (adult/juvenile)^c^	Sample size blood smear	Flyway
Egypt	Lake Nasser	TD^d^	2019	BS	9 (5/4)	9	-
		LD	2019	-	4 (4/0)	4	-
		CD	2019	-	1 (0/1)	1	-
		ND	2019	-	1 (1/0)	1	-
France	Chizé	TD	2014	BS	5 (X/X)^e^	5	West
	Île d'Oléron	TD	2014	BS	34 (X/X)	34	West
Germany	Brandenburg	TD	2018/19	BS	4 (4/0)	4	Central/east
	Saxony	SD	2013	BS	2 (2/0)	2	-
	Hesse	TD	2014/18/19	BS	7 (7/0)	7	West
		SD	2013/14/18/19	BS	50 (50/0)	49	-
		WP	2018/19	Year-round	15 (14/1)	14	-
		WP VetMed^f^	2019	Year-round	45 (9/36)	44	-
Greece	Soufli	TD	2015	BS	3 (0/3)	3	Central/east
	Antikythira Island	TD	2018/19	AM/SM	3/46 (48/1)	47	Central/east
Italy	Ventotene Island	TD	2014	SM	27 (27/0)	24	Central/east
Malta	Comino Island	TD	2014	SM	2 (2/0)	2	Central/east
Spain	National Park Monfragüe	TD	2013	BS	1 (X/X)	1	West

^a^TD = European turtle dove *Streptopelia turtur*, LD = laughing dove *S. senegalensis*, CD = collared dove *S. decaocto*, ND = Namaqua dove *Oena capensis*, SD = stock dove *Columba oenas*, WP = common woodpigeon *C. palumbus*

^b^BS = breeding season (sampled June to August), SM = spring migration (sampled April to May) and AM = autumn migration (sampled in September)

^c^Juvenile = hatched during the current calendar year. No nestlings were included

^d^Subspecies *S*. *t*. *rufescens* (Brehm 1845). All other sampled turtle doves belong to the nominate subspecies *S*. *t*. *turtur* (Linnaeus 1758)

^e^‛X’ is given when no information about the age was available

^f^Woodpigeons were brought to the Clinic for Birds, Reptiles, Amphibians and Fish in Giessen by the public

### Parasite detection

#### Nested PCR assay and Sanger sequencing

The presence or absence of avian haemosporidians was determined through nested polymerase chain reaction (PCR) targeting a 479 base pair (bp) region of the cytochrome *b* gene (cyt *b*; Hellgren et al. 2004). For the initial PCR reaction, the primer pair HaemNFI/HaemNR3 was applied. A 4 μl aliquot of this PCR product was subsequently used as template DNA for the second PCR reactions with specific primer pairs HaemF/HaemR2 for *Haemoproteus* (henceforth *Haemoproteus* refers to both subgenera *H*. (*Haemoproteus*) and *H*. (*Parahaemoproteus*) infections, unless explicitly defined) and *Plasmodium* and HaemFL/HaemR2L for *Leucocytozoon* amplification. All PCR reactions were carried out in a 25 μL reaction volume containing 12.5 μl 2x DreamTaq Master-Mix (Thermo Fisher Scientific, USA), 1.65 μl of each primer (10 μM), 4 μl template DNA (20–80 ng/μl) and 5.2 μl deionized water. DNA from passerine birds with known infection and deionized water were included in each PCR run as positive and negative controls, respectively. PCR protocols (see Hellgren et al. 2004 for cycling conditions) were carried out on a Biometra TOne Cycler (Analytik Jena, Germany).

As multiple PCR runs can produce additional positives (Dunn et al. 2017), each sample resulting in a negative PCR reaction was conducted a second time to confirm the absence of parasites, whereas a single positive PCR result was interpreted as an infected bird. PCR products of samples rendering a clear band during gel electrophoresis (QIAxcel Advanced, Qiagen, Switzerland) were Sanger sequenced bi-directional by Microsynth-Seqlab (Sequence Laboratories Goettingen GmbH, Germany). Forward and reverse sequences were assembled and trimmed in CLC Main Workbench 7.6.4 (CLC Bio, Qiagen, Denmark) and checked for mixed infections (Ferreira Junior et al. 2017).

To identify lineages, the sequences were aligned with reference sequences deposited in MalAvi database (Bensch et al. 2009) using BLASTN 2.3.0+ (Zhang et al. 2000). Sequences are considered as distinct lineages if they differ by one or more nucleotides in the cyt *b* fragment (Hellgren et al. 2004; Bensch et al. 2009). Lineages with no database records in MalAvi were considered novel. For novel lineages, PCR and sequencing were performed twice to verify the results. Novel sequences and sequences found in a host species for the first time are deposited in GenBank under accession numbers MT888848-60.

#### One-step multiplex PCR assay

The aforementioned widely used nested PCR assay is sufficient for genus and lineage identification. However, it is ineffective at detecting mixed infections of *Haemoproteus* and *Plasmodium* because it favours the amplification of the most abundant parasite in the sample or the parasite for which the primers are a better match (Ciloglu et al. 2019). But since mixed infections are very common and have been shown to be particularly virulent (Valkiūnas et al. 2006; Bernotienė et al. 2016), a PCR assay for simultaneous detection of *Plasmodium*, *Haemoproteus* and *Leucocytozoon* was additionally applied for samples tested positive for either *Haemoproteus* or *Plasmodium* by the nested PCR assay.

The PCR was performed according to Ciloglu et al. (2019) by using equimolar concentrations of three primer sets PMF/PMR, HMF/HMR and LMF/LMR in a single reaction tube, targeting different sized fragments (approx. 380 bp fragment of non-coding region of *Plasmodium* mtDNA, approx. 530 bp fragment between the 5′ end of cyt *b* and a non-coding region of mtDNA of *Haemoproteus*, and approx. 220 bp fragment of the cytochrome *c* oxidase subunit 1 (COX1) gene of *Leucocytozoon*, respectively).

The reactions were set up in total volumes of 20 μl containing 10 μl of 2x Multiplex PCR Master-Mix (Qiagen, Hilden, Germany), 0.4 μl of each primer (10 μM), 3.6 μl of deionized water and 4 μl of DNA template. PCR protocols (see Ciloglu et al. 2019 for cycling conditions) were carried out on a Biometra TOne Cycler. Every PCR run contained positive and negative samples (cf. nested PCR assay). PCR amplicons were visualized using QIAxcel Advanced (Qiagen, Switzerland) high-resolution capillary gel electrophoresis.

#### Examination of blood films

To confirm the presence or absence of intracellular parasite gametocytes, blood smears (*n* = 251) were examined at × 1000 magnification for at least 10,000 monolayered erythrocytes using a light microscope (PrimoStar Zeiss, Germany). The intensity of parasitemia was determined by counting the number of infected blood cells per 10,000 erythrocytes (Godfrey et al. 1987). Identification of haemosporidian parasites, limited to genus level, followed the criteria of Clark et al. (2009).

### Phylogenetic and statistical analyses

Constructions of lineage networks, using the medium joining network method, were performed with PopART 1.7 (Leigh and Bryant 2015).

For phylogenetic tree reconstruction, in addition to newly found lineages, we downloaded one sequence from NCBI GenBank for each haemosporidian lineage (*n* = 148) shown to infect species of the order Columbiformes and deposited in MalAvi (MalAvi 2020). Some sequences (*n* = 45) were excluded due to insufficient sequence length or only partial coverage of the chosen 477 bp cyt *b* fragment. However, all lineages detected in the present study are represented in the phylogenetic analysis.

The best-fit model of DNA sequence evolution was selected using jModeltest 2.1.7 (Darriba et al. 2012). According to the Akaike information criterion, we used the General Time Reversible model including invariable sites and variation among sites (GTR+I+G; Gu et al. 1995). Phylogenetic reconstruction was performed with BEAST 1.8.4. (Drummond et al. 2012). Tree priors were selected using the interface BEAUTi 1.8.4. with strict clock and a Yule speciation process (Yule 1925; Gernhard 2008). Markov chain Monte Carlo (MCMC) simulations were run with 50,000,000 generations and one tree was recorded every 1000 generations. In all, 10% of the trees were discarded as burn-in in TreeAnnotator (BEAST package). We validated the results of the Bayesian analyses in Tracer 1.6. (Drummond and Rambaut 2007). The phylogenetic tree was constructed with FigTree 1.4.3 (Rambaut 2007).

Statistical analyses were performed with R 3.6.3 (R Core Team 2016). Due to a sufficient sample size (cf. Jovani and Tella 2006), we selected turtle doves (*n* = 141), woodpigeons (*n* = 60) and stock doves (*n* = 52) to assess whether overall prevalence and prevalence per genus varied across species. To compare the equality of proportions (e.g. to assess the difference in prevalence between species), the frequency distribution test ‘Pearson’s chi-squared test’ was applied. To determine whether prevalence (infection status as determined by nested PCR expressed as binominal contrast: presence/absence of infection) of turtle doves was associated with timing of sampling (breeding season vs. spring migration) or flyway, we constructed a general linear model (GLM). A significance level of *p* < 0.05 was used.

## Results

### Prevalence of haemosporidian parasites

Of the 259 individuals screened for haemosporidian parasites using specific nested PCR assay, 109 were infected (42.1%). We successfully obtained a sequence from all positive PCR results (*n* = 109). Most individuals (*n* = 40) were infected with a single *Leucocytozoon* lineage (15.4%), 40 with a single *Haemoproteus* lineage (15.4%; divided in *H.* (*Haemoproteus*) 13.1%, *H.* (*Parahaemoproteus*) 2.3%) and nine with a single *Plasmodium* lineage (3.5%). Two individuals (0.8%) had mixed homogeneric infections, 17 columbiform birds (6.6%) showed different types of heterogeneric infections and one turtle dove had both (*Haemoproteus*/*Haemoproteus*/*Leucocytozoon*) (Table 2).

**Table 2:** Number of avian haemosporidian infections per sampled species and sites. The composition of occurring mixed infections is shown in the right-most column. We refer ‘infected’ birds to individuals tested positive through nested PCR assay

Host species^a^	Sampling location	Total	Prevalence [%]^b^	INF	H^c^	P	L	M	Mixed
TD	Egypt	9	33.3	3	3	0	0	0	
	France	39	15.4	6	2	4	0	0	
	Germany	11	45.5	5	4	1	0	0	
	Greece	52	75.0	39	22	2	3	12	10HL, 1PL, 1HHL
	Italy	27	55.6	15	4	1	8	2	1HL, 1LL
	Malta	2		1	1	0	0	0	
	Spain	1		0	0	0	0	0	
	**Total TD**	141	48.9	69	36	8	11	14	11HL, 1PL, 1HHL, 1LL
WP	Germany	60	61.7	37	2	0	29	6	5HL, 1LL
SD	Germany	52	3.8	2	1	1	0	0	
ND	Egypt	1		1	1	0	0	0	
LD	Egypt	4		0	0	0	0	0	
CD	Egypt	1		0	0	0	0	0	
**Overall total**		**259**	**42.1**	**109**	**40**	**9**	**40**	**20**	

^a^TD = European turtle dove *Streptopelia turtur*, LD = laughing dove *S*. *senegalensis*, CD = collared dove *S*. *decaocto*, ND = Namaqua dove *Oena capensis*, SD = stock dove *Columba oenas*, WP = common woodpigeon *C*. *palumbus*

^b^Prevalence is only given if the sample size was ≥ 5

^c^H = *Haemoproteus* (subgenera *H*. (*Haemoproteus*) and *H*. (*Parahaemoproteus*) combined), P = *Plasmodium*, L = *Leucocytozoon*, M = mixed infections

**Fig. 1:**
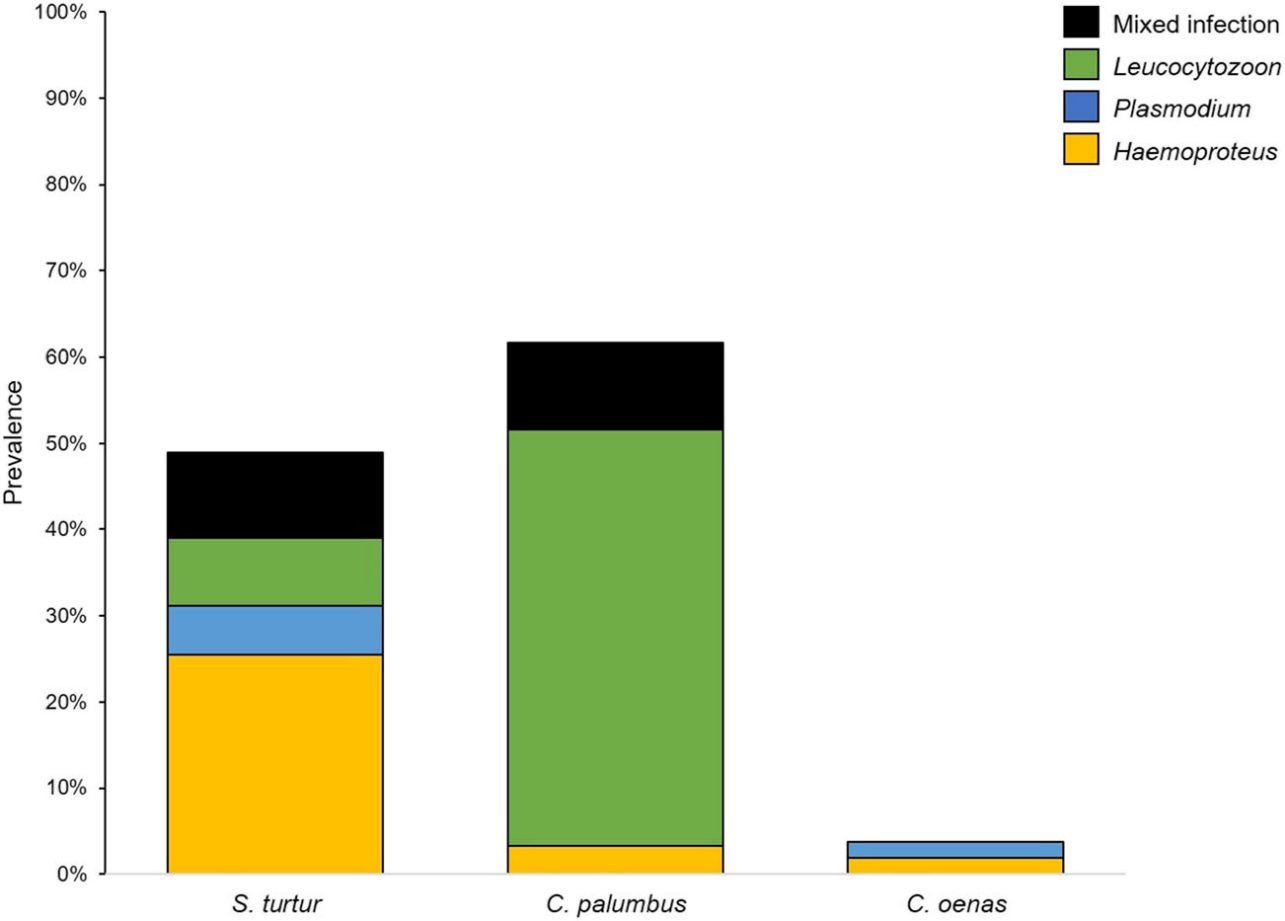
Prevalence of infection [%] for haemosporidian genera (*Haemoproteus* refers to the subgenera *H*. (*Haemoproteus*) and *H*. (*Parahaemoproteus*) combined) and mixed infections in the three species: European turtle doves *Streptopelia turtur* (*n* = 141), common woodpigeons *Columba palumbus* (*n* = 60) and stock doves *C. oenas* (*n* = 52). We refer ‘infected’ birds to individuals tested positive through nested PCR assay

Woodpigeons had the highest overall prevalence (61.7%), followed by turtle doves (48.9%), while stock doves had the lowest prevalence (3.8%) (Fig. 1). Overall prevalence differed significantly between the species (*χ*^2^ = 43.2, df = 2, *p* < 0.001). Comparing the species pairwise, there was a significant difference between stock doves and the other two species (stock dove/woodpigeon *χ*^2^ = 41.0, df = 1, *p* < 0.001; stock dove/turtle dove *χ*^2^ = 33.2, df = 1, *p* < 0.001), while there was no significant difference between turtle doves and woodpigeons (*χ*^2^ = 2.7, df = 1, *p* = 0.098). In turtle doves, a single infection with *Haemoproteus* was the most prevalent (25.5%), whereas in woodpigeons, it was a single infection with *Leucocytozoon* (48.3%). Turtle doves were significantly more often infected with *Haemoproteus* compared to woodpigeons (*χ*^2^ = 10.6, df = 1, *p* = 0.001) and stock doves (*χ*^2^ = 20.7, df = 1, *p* < 0.001). Conversely, woodpigeons significantly showed more infections with *Leucocytozoon* than turtle doves (*χ*^2^ = 33.1, df = 1, *p* < 0.001) and stock doves (*χ*^2^ = 44.1, df = 1, *p* < 0.001). An infection with *Plasmodium* was present at a rather low frequency in turtle doves (5.6%) and stock doves (1.9%) and absent in woodpigeons. *Haemoproteus* and *Plasmodium* infections were found in only one stock dove individual each (1.9%). We did not detect *Leucocytozoon* infections in any sampled stock doves. Mixed infection occurred in turtle doves and woodpigeons with a similar prevalence (9.9% and 10%, respectively) (Fig. 1).

There was no age-related significant difference (juveniles vs. adults) in woodpigeons for neither *Haemoproteus* (*χ*^2^ = 0.9, df = 1, *p* = 0.334) nor *Leucocytozoon* infections (*χ*^2^ = 1.3, df = 1, *p* = 0.256).

In turtle doves, an infection with *Haemoproteus* and *Leucocytozoon* depended on the sampling season, while there was no effect of the migration flyway (*Haemoproteus*: season: *χ*^2^ = 4.0, df = 1, *p* = 0.045; flyway: *χ*^2^ = 0.1, df = 1, *p* = 0.781; *Leucocytozoon*: season: *χ*^2^ = 5.1, df = 1, *p* = 0.024; flyway: *χ*^2^ < 0.001, df = 1, *p* = 1.0). Independent of sampling location in Europe, *Haemoproteus* was most prevalent in turtle doves sampled during spring migration (*n* = 75; 52.0%) compared to breeding season (*n* = 54; 11.1%) and autumn migration (*n* = 3; 0.0%). *Leucocytozoon* infections (32.0%) could be detected for individuals sampled during spring migration only. *Plasmodium* infections were neither dependent on flyway nor sampling season (season: *χ*^2^ = 0.7, df = 1, *p* = 0.392; flyway: *χ*^2^ = 1.5, df = 1, *p* = 0.227).

### Lineage diversity and phylogenetic analyses

**Fig. 2:**
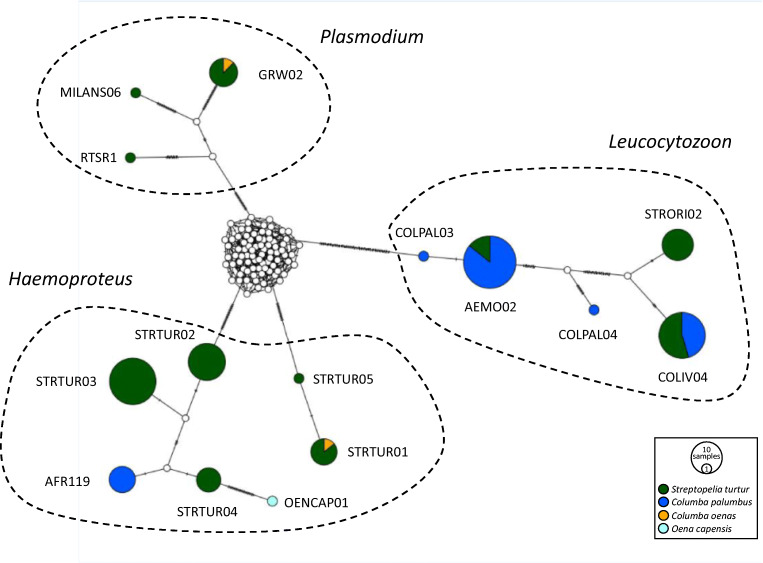
Median-joining network of mitochondrial cytochrome *b* gene lineages (*n* = 130, 496 bp fragment) of haemosporidian parasites *Haemoproteus* (refers to the subgenera *H*. (*Haemoproteus*) and *H*. (*Parahaemoproteus*) combined), *Leucocytozoon* and *Plasmodium* infecting columbiform birds. Circles represent lineages, and the circle sizes are proportional to the lineage frequencies in the sample set. Lineage names are noted at the associated circles. One hatch mark represents one mutation. Sampled host species are represented by different colours

The 109 positive samples represented 15 distinct lineages, including five *H*. (*Haemoproteus*), two *H*. (*Parahaemoproteus*), three *Plasmodium* and five *Leucocytozoon* lineages (Fig. 2, Table 3). The highest lineage diversity was found in turtle doves (*n* = 11), followed by woodpigeons (*n* = 5) and stock doves (*n* = 2). The most frequently occurring lineage was AEMO02 (*Leucocytozoon*), followed by COLIV04 (*Leucocytozoon*) and the *H*. (*Haemoproteus)* lineages STRTUR02 and STRTUR03. All other lineages had a prevalence of less than 10% (Table 3). Eleven lineages were present in a single host species, whereas the remaining four infected two host species (Fig. 2).

**Table 3:** Haemosporidian lineages found in bird species of the order Columbiformes with the respective GenBank accession number and lineage prevalence. If mixed homogeneric infection occurred, the lineage combination of the two lineages is presented

Parasite genus^a^	Lineage (MalAvi)	Accession number (GenBank)	Host species^b^ (no. infected individuals)	First time record for^c^	Lineage prevalence (%)^d^	Homogeneric mixed with
H	AFR119	KM056425	WP (7)^#^		5.4	-
H	OENCAP01*	MT888850	ND (1)^#^		0.8	-
ParaH	STRTUR01	KJ488784	TD (6)^#^ SD (1)^#^	SD	5.4	-
H	STRTUR02	KJ488786	TD (14)^#^		10.8	STRTUR04
H	STRTUR03	KJ488826	TD (22)^#^		16.9	-
H	STRTUR04*	MT888848	TD (6)^#^		4.6	STRTUR02
ParaH	STRTUR05*	MT888849	TD (1)^#^		0.8	-
L	AEMO02	KJ488804	TD (4)^#^ WP (24)^#^	TD	21.5	COLIV04 STRORI02
L	COLIV04	AB741510	TD (12)^#^ WP (10)^#^	TD WP	16.9	AEMO02
L	COLPAL03*	MT888851	WP (1)		0.8	-
L	COLPAL04*	MT888852	WP (1)^#^		0.8	-
L	STRORI02	AB741508	TD (10)^#^	TD	7.7	AEMO02
P	GRW02	AF254962	TD (7)^#^ SD (1)^#^	TD SD	6.2	-
P	MILANS06	JN164715	TD (1)	TD	0.8	-
P	RTSR1	KJ488785	TD (1)		0.8	-

^a^H = *H*. (*Haemoproteus*), ParaH = *H*. (*Parahaemoproteus*), L = *Leucocytozoon*, P = *Plasmodium*

^b^TD = European turtle dove *Streptopelia turtur*, LD = laughing dove *S*. *senegalensis*, CD = collared dove *S*. *decaocto*, ND = Namaqua dove *Oena capensis*, SD = stock dove *Columba oenas*, WP = Common woodpigeon *C*. *palumbus*

^c^According to MalAvi (2020)

^d^Percentage of each lineage among all lineage sequences (*n* = 130)

*Novel lineages found in the present study

^#^Lineages present during microscopic examination (infected erythrocytes)

We identified five novel lineages. Novel lineages were found in turtle doves (STRTUR04 *H*. (*Haemoproteus*), MT888848 and STRTUR05 *H*. (*Parahaemoproteus*), MT888849) and in the Namaqua dove sample (OENCAP01 *H*. (*Haemoproteus*), MT888850). Two novel *Leucocytozoon* lineages infected woodpigeons (COLPAL03 MT888851 and COLPAL04 MT888852). For some already known lineages, we provide first records of them infecting species of the order Columbiformes (Table 3).

**Fig. 3:**
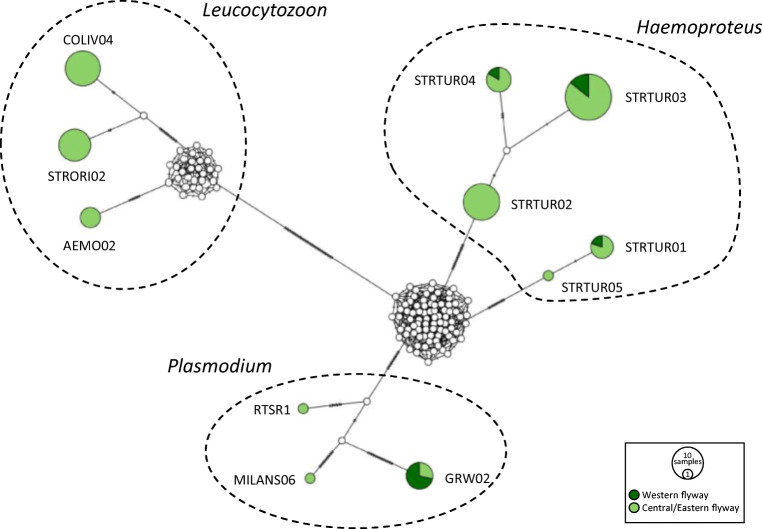
Median-joining network of mitochondrial cytochrome *b* gene lineages (*n* = 81, 496 bp fragment) of haemosporidian parasites *Haemoproteus* (refers to the subgenera *H*. (*Haemoproteus*) and *H*. (*Parahaemoproteus*) combined), *Leucocytozoon* and *Plasmodium* infecting European turtle doves *Streptopelia turtur*. Circles represent lineages, and the circle sizes are proportional to the lineage frequencies in the sample set. Lineage names are noted at the associated circles. One hatch mark represents one mutation. Positive samples of individual birds from the western (*n* = 10 sequences) and the central/eastern flyway (*n* = 71 sequences) are represented by different colours

Turtle doves following the central/eastern flyway showed a higher lineage diversity for all haemosporidian genera than individuals using the western flyway (*Haemoproteus*: five vs. three, *Plasmodium*: three vs. one, and *Leucocytozoon*: three vs. zero lineages, respectively). All lineages infecting turtle doves sampled along the western flyway were also found in individuals following the central/eastern flyway (Fig. 3).

**Fig. 4:**
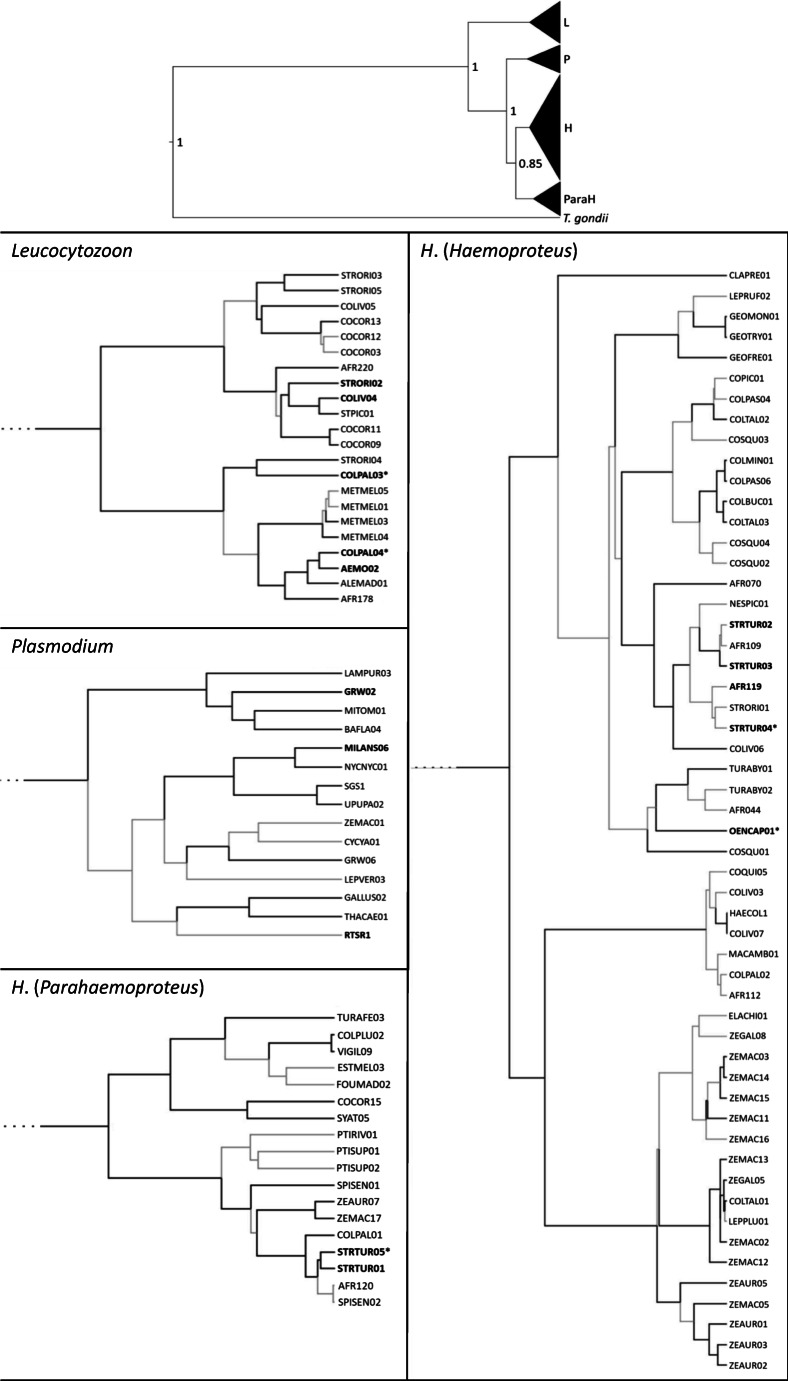
Phylogeny of mitochondrial cytochrome *b* gene lineages (477 bp fragment) of avian haemosporidian parasites (*n* = 109) isolated from blood samples of columbiform birds based on a Bayesian analysis. The four different clades *Leucocytozoon* (L), *Plasmodium* (P), *H*. (*Haemoproteus*) (H) and *H*. (*Parahaemoproteus*) (ParaH) are shown in the overview tree and in one individual zoomed-in tree each together with lineage names. Lineages found in the present study are shown in bold. Newly discovered lineages are marked with ‘*’. *Toxoplasma gondii* (KM657812) was included as outgroup. GenBank accession numbers are given in Table S1. Nodal support values indicate posterior clade probabilities. If the node support is not shown by digits, nodes with posterior probabilities < 90% are coloured in grey

The Bayesian-based phylogeny of mitochondrial cyt *b* gene fragment revealed three well-supported major clades, representing *Leucocytozoon*, *Plasmodium* and *Haemoproteus*. Whereby *Haemoproteus* showed two monophyletic subclades, indicating the subgenera *H*. (*Parahaemoproteus*) and *H*. (*Haemoproteus*) (Fig. 4). From the 109 lineages included in the phylogenetic tree reconstruction, 22 belong to *Leucocytozoon*, 15 to *Plasmodium* and 72 to *Haemoproteus*, divided in 54 lineages clustering to *H*. (*Haemoproteus*) and 18 to *H*. (*Parahaemoproteus*).

### Blood slide screening and one-step multiplex PCR assay

The overall prevalence according to counts of infected erythrocytes in blood smears was 28.3% (*Haemoproteus* 12.7%, *Plasmodium* 1.2%, *Leucocytozoon* 4.8%; 27 samples could not be assigned reliably to one of the genera). This overall prevalence is lower than the prevalence according to nested PCR results (42.1%) (*χ*^2^ = 10.6, df = 1, *p* = 0.001). With the exception of COLPAL03, MILANS06 and RTSR1, we found in at least one sample per lineage infected erythrocytes (Table 3).

The average parasitemia estimated from blood smears was 17.6, ranging from 0.8 to 912.3 parasites per 10,000 erythrocytes. However, 90.1% of the samples had a parasitemia lower than 10 infected blood cells per 10,000 erythrocytes. One turtle dove sampled during spring migration in 2019 in Greece showed an extremely high parasitemia with 912.3 parasites per 10,000 erythrocytes (Fig. S2) compared to the remaining samples (maximum of 43.6 parasites per 10,000 erythrocytes).

From the 259 samples, 67 were tested positive for *Haemoproteus* or *Plasmodium* with the nested PCR assay. These samples were included in the one-step multiplex PCR runs. The one-step multiplex PCR assay showed a positive PCR result for 64 (95.5%) samples. All *Plasmodium* lineages GRW02, MILSAN06 and RTSR1 were displayed at the expected band height. While *Haemoproteus* lineages STRTUR01 and STRTUR05 were displayed at the expected band height, AFR119, STRTUR02, STRTUR03 and STRTUR04 were displayed at a height expected for *Plasmodium* lineages. All *Haemoproteus* lineages with ‘wrong’ band heights cluster in the phylogenetic tree into the *H*. (*Haemoproteus*) clade (Fig. 4) and group together in the networks (Figs. 2 and 3). An apparent mixed infection of *Haemoproteus* and *Plasmodium* was present in three turtle dove samples according to the results of the one-step multiplex PCR assay. No mixed *Haemoproteus*/*Plasmodium* infection could be proved by the nested PCR assay (Table 2) and for one turtle dove only by microscopic examination. However, for the mixed infections according to the one-step multiplex PCR assay, we cannot distinguish between *Plasmodium*/*Haemoproteus* mixed infections and mixed infections of the two subgenera *H*. (*Haemoproteus*) and *H*. (*Parahaemoproteus*) due to the ‘wrong’ band heights displayed for *H*. (*Haemoproteus*) lineages. Given these inconsistencies, we classified individuals tested positive through nested PCR-based assay (and verified by Sanger sequencing) as ‘infected’.

## Discussion

### Interspecific differences and lineage diversity

Even though common lineages were detected in the sampled columbiform birds, the overall and genus-specific prevalence as well as the lineage diversity differed among the species. Similar differences in parasite prevalence were reported from other closely related species (e.g. tree sparrow *Passer montanus* and house sparrow *P. domesticus*, Lee et al. 2006; great tit *Parus major* and blue tit *Cyanistes caeruleus*, Dubiec et al. 2016).

Our results could have been affected by changes in prevalence over time, as the samples were collected from various years. However, there are studies that found no significant changes in the parasite or lineage prevalence between years (Bensch et al. 2007; Shurulinkov and Ilieva 2009, but see Dunn et al. 2017) and the majority of studies investigating prevalence are based on non-uniform distributed data (e.g. birds of different age or variable sample sizes among sample locations; see Dubiec et al. 2016). Therefore, we deem at least our observed main patterns of prevalence and lineage diversity as reliable and evaluable.

The general pattern with woodpigeons showing the highest overall prevalence, followed by turtle doves and stock doves being the least infected ones, is quite similar with the results of Dunn et al. (2017), who tested nestlings of these species. However, in contrast to Dunn et al. (2017), we found evidence for *Plasmodium* infections in turtle doves and stock doves. The fact that woodpigeons and turtle doves showed a significantly higher infection rate than stock doves might be due to different nesting behaviours. Open-nesting species are expected to have higher rates of infection than cavity-nesters (Hellard et al. 2016; Dunn et al. 2017, but see Quillfeldt et al. 2011). While turtle doves and woodpigeons built open nests in shrubs or trees, stock doves breed in tree cavities or artificial nest boxes (von Blotzheim and Bauer 1994). We found the overall prevalence was markedly higher in open-nesting vs. cavity-nesting species. Since only one stock dove individual each was infected with *Haemoproteus* or *Plasmodium*, it is possible that also *Leucocytozoon* infections occur in stock doves at a similar low rate, even if we could not prove this in our study. Dunn et al. (2017) could prove an infection with *Leucocytozoon* for a single stock dove nestling. The nesting behaviour together with other behavioural traits (e.g. being less gregarious or having a low habitat overlap with other columbiform birds, particularly feral pigeons) may explain the low prevalence in stock doves. However, also other factors such as differences in the host immune system, resistance to parasites and other idiosyncrasies of vectors and parasites (Reinoso-Pérez et al. 2016) may influence the low infection rates in stock doves.

*Haemoproteus* is the most frequently reported blood parasite in birds, followed by *Leucocytozoon* and *Plasmodium* (Carlson et al. 2013; Heym et al. 2019). In this study, this pattern was observed for turtle doves, but not for woodpigeons, for which *Leucocytozoon* was the most prevalent. Although the *Leucocytozoon* prevalence was rather high (58.3%), the genetic diversity was rather low. The two lineages AEMO02 and COLIV04 were present in 94.4% of *Leucocytozoon*-infected woodpigeons. Both lineages were detected in feral pigeons previously (MalAvi 2020). Feral pigeons are widely distributed and their number is increasing, especially in urban areas (Haag-Wackernagel and Moch 2004). In this study, the majority of sampled woodpigeons (82%) were from urban areas. Hence, it is likely that feral pigeons could have acted as a reservoir and blackflies transmitted the lineages from feral pigeons to woodpigeons and vice versa. While Scaglione et al. (2015) states that *Leucocytozoon* parasites are not routinely found in pigeons, a study sampling blackflies in Central Europe showed pigeons to be one of the main targets of ornithophilic blackflies (Chakarov et al. 2020). High rates of *Leucocytozoon* in the sampled woodpigeons may have been favoured by the opportunities for transmission that the woodpigeons offered (e.g. flocking behaviour, increased host densities in urban areas, proximity to feral pigeons; cf. Sol et al. 2000).

High infection rate with *Leucocytozoon* and lower rates of *Plasmodium* and *Haemoproteus* in residents or short-distance migrants (woodpigeons) compared to higher Haemoproteidae prevalence in long-distance migrants (turtle doves) were observed. This is in line with previous research, as Haemoproteid transmission to the birds breeding in the northern hemisphere takes place mostly at wintering areas and along the migration route of the long-distance migrants in contrast to *Leucocytozoon* that is transmitted mainly at the breeding grounds (Valkiūnas 2005; Shurulinkov and Ilieva 2009).

Turtle doves hosted the highest parasite diversity, being the only species infected with all tested haemosporidian genera and harbouring the highest number of lineages. This is in line with previous studies, which have shown that migratory birds have a higher prevalence and diversity of blood parasites than resident or short-distance migratory species (Figuerola and Green 2000; Jenkins et al. 2012; Walther et al. 2016; Ciloglu et al. 2020b). This could be explained by the fact that residents and short-distance migrants travel between areas that are likely to be within a single transmission area, e.g. within a continent, and so are confronted with a single parasite fauna (Hellgren et al. 2007; Jenkins et al. 2012). In contrast, long-distance migrants move between vastly separated areas and thereby encounter different faunas of parasites (Waldenström et al. 2002). Being a long-distance migrant also increases the time being exposed to parasites compared to residents or short-distance migrants in temperate regions, which lack parasite transmission during autumn and winter when vector activity wanes (Cosgrove et al. 2008). Furthermore, environmental challenges, inducing stress and increased day length at the distant wintering grounds may induce infections (Valkiūnas et al. 2004). Since migration is an energetically costly, strenuous physical activity, resources may be traded-off from immune defence, making it likely that migrant species are more susceptible than residents (Waldenström et al. 2002; Jenkins et al. 2012, but see Hegemann et al. 2012).

Migratory birds can transport parasite lineages to novel environments (Waldenström et al. 2002; Adamik et al. 2016). It has been suggested that some lineages are transmitted in Africa only, while others are transmitted in Europe only and a few in both continents (Ferraguti et al. 2019). The presence of the seven lineages found in woodpigeons and stock doves (Table 3) indicates that these lineages are transmitted in Europe. For turtle doves of the subspecies *S*. *t*. *turtur* as Palearctic-African migrants, transmission can take place both on the African and European continents, whereas for the subspecies *S*. *t*. *rufescens*, infected with the lineages STRTUR01, STRTUR02 and STRTUR03, transmission can occur in Africa only. As for some lineages (COLPAL03, MILSAN06 and RTSR1), no infected erythrocytes could be found during blood smear screening and possible mixed infections of lineages belonging to the same genus were not considered in the microscopic examinations, assigned transmission areas are likely but not definitely proven.

Despite a rather high overall prevalence, the infection intensity (parasitemia) was in general rather low. Studies on parasitemia are still limited despite their importance (Huang et al. 2020). The damage produced on the host species greatly depends on the infection intensity with the harmful effects being most pronounced when parasitemia is very high (Sol et al. 2003). However, medication experiments have shown that also chronic infections at lower intensities can influence host reproductive success and conditions (Merino et al. 2000; Knowles et al. 2010). To our knowledge, there is no study examining the parasitemia of the species sampled here. However, the average parasitemia (approx. 10 parasites per 10,000 erythrocytes) was lower than in wild columbiform birds sampled in Nigeria (100 per 10,000; Akinpelu 2008), on the Canary Islands (148 per 10,000; Foronda et al. 2004) and in India (*Haemoproteus*: 350, and *Plasmodium* 150 per 10,000; Gupta et al. 2011). Only one of our sampled birds, a turtle dove, showed a severe *Haemoproteus* infection with parasitemia of approx. 900 parasites per 10,000 erythrocytes. However, the adverse effects of haemosporidian infections to the avian host depend on many factors (e.g. host immunity, food availability, or infection intensity) (Chagas et al. 2016) and are therefore difficult to assess for our sampled birds.

### Intraspecific differences in turtle doves

Population genetic analyses have shown that turtle doves are not genetically structured across their flyways (Calderon et al. 2016). For haemosporidian parasites infecting turtle doves, we found differences in *Haemoproteus* and *Leucocytozoon* prevalence with the timing of sampling, but no significant differences in prevalence between the flyways. These results are consistent with other studies that have described seasonal variation in the prevalence over the annual cycle (Klei and DeGiusti 1975; Cosgrove et al. 2008; Hellgren et al. 2013; Dubiec et al. 2016; Walther et al. 2016; Pulgarín-R et al. 2018; Soares et al. 2020).

*Leucocytozoon* prevalence was highest in turtle doves sampled during spring migration. This pattern fits previous work showing that *Leucocytozoon* infections mainly occur in spring and autumn (Atkinson and van Riper 1991). However, other studies found contrasting *Leucocytozoon* prevalence patterns. Significantly, lower infection rates at spring migration stopover sites compared to breeding areas were detected for redstarts *Phoenicurus phoenicurus* (Rintamäki et al. 1999). In garden warblers *Sylvia borin*, pooled *Leucocytozoon* infections showed no circannual variation in prevalence, but variation could be detected for some lineages when analysed individually (Hellgren et al. 2013). The higher infection rate of *Leucocytozoon* in turtle doves in spring might be due to a seasonal outbreak. The infection peak might be induced by a spring relapse due to physiological cues in the host (Applegate and Beaudoin 1970; Valkiūnas et al. 2004; Cornelius et al. 2014) and/or with the return of the simuliid vectors (adult female blackflies) in spring, when environmental conditions enable increased blackfly emergence and activity (Reidelbach and Christl 2002). Mechanisms for seasonal peaks in infection outbreaks include changes in the behaviour and physiology of the parasite, vector and host, but these are difficult to tease apart as many of these changes occur simultaneously (Cornelius et al. 2014). *Haemoproteus* prevalence in turtle doves was particularly high during migration compared to the breeding season. Klei and DeGiusti (1975) determined a peak of *H. columbae* infection in feral pigeons during autumn; other studies demonstrated *Haemoproteus* peaks not only during autumn migration but also during the breeding season (Hellgren et al. 2013; Pulgarín-R et al. 2018). Besides sampling date, *Haemoproteus* prevalence and diversity might be influenced by differences at the African wintering grounds, where *Haemoproteus* transmission mainly takes place (Valkiūnas 2005; Waldenström et al. 2002; Shurulinkov and Ilieva 2009). Mirroring the different migration flyways of turtle doves, studies indicate different winter regions in the western, central and eastern Sub-Sahara (Zwarts et al. 2009). Pathogen transmission may be an important driver of site selection during the non-breeding period. Some migration strategies are thought to be the result of species actively avoiding parasite-rich habitats by choosing a winter site with low prevalence of haemosporidian parasites (Waldenström et al. 2002; Clark et al. 2016). However, many variables shape migration patterns and non-breeding habitat choice, and the relative importance of active parasite avoidance compared to other factors needs further research (Clark et al. 2016).

*Plasmodium* infections showed no seasonality in our study. Contrarily, seasonal variation in pooled *Plasmodium* prevalence was found in a population of blue tits with prevalence peaks in spring and autumn. However, this variation was present in pooled *Plasmodium* infections only, whereas *P*. *relictum* prevalence was more stable through the annual cycle (Cosgrove et al. 2008). In garden warblers, *Plasmodium* infection peak was during the wintering stage. However, this pattern was not consistent for all lineages, and one of the most common *Plasmodium* lineages SGS1 showed no significant circannual variation (Hellgren et al. 2013). To give a more accurate picture of genus-specific or even lineage-specific seasonality of haemosporidian parasites in turtle doves, we would need samples from the autumn migration and wintering season.

Haemosporidian parasite prevalence was rather high in turtle doves and these parasites can have negative effects on hosts, but as observed parasitemia was rather low, we deem the contribution of haemosporidian infections to the turtle doves’ decline to be rather insignificant. *Plasmodium* is known to cause mortality in wild susceptible bird populations, especially when birds are co-infected with other pathogens such as Usutu virus (Rouffaer et al. 2018). However, *Plasmodium* infection in turtle doves was rather rare and the impact of *Haemoproteus* and *Leucocytozoon*, which had a higher prevalence, on avian populations is generally thought to be less severe (Yanga et al. 2011). Nevertheless, the future development of avian haemosporidians in declining turtle doves should be monitored. Under a scenario of global change, a temperature increase and anthropogenic land-use change may provide new opportunities for blood parasite transmission in areas where they were previously absent as well as alter their diversity and composition (Dunn and Outlaw 2019; Ferraguti et al. 2019; Heym et al. 2019).

The identification of current transmission areas and parasite diversity is highly relevant to recognize and understand possible future changes (Ciloglu et al. 2020b). As habitat destruction and land-use intensification are among the main reasons causing the sharp decline of turtle doves (Fisher et al. 2018), they seem especially prone to be affected by these changes, and thus, parasites could gain importance as threats in the future.

### Methodological inconsistencies regarding parasite prevalence

To achieve an assessment of prevalence as accurately as possible, we applied two PCR assays as well as microscopic examination. Different methodological approaches led to differing prevalence. A lower prevalence was derived from blood smear counts. This is in line with other studies on columbiform birds, determining a lower prevalence based on microscopic examination compared to molecular techniques (Dunn et al. 2017; Tavassoli et al. 2018). The absence of gametocytes in blood smears of birds PCR positive can be explained by light gametocyte parasitemia, DNA amplification of circulating sporozoites or presence of remnants of parasites that aborted development (Valkiūnas et al. 2009; Chagas et al. 2016). The PCR-based method displays the detection of parasitic genome, but does not reveal whether parasites have or will develop into a successful infection (Valkiūnas 2005). Therefore, microscopic examination is important to distinguish between abortive and successful chronic infections. In general, when finding a ‘rare’ lineage, we cannot exclude the scenario that the host is a ‘dead-end’ (Hellgren et al. 2013). For three ‘rare’ lineages (COLPAL03, MILANS06, RTSR1), we could not find infected erythrocytes, indicating a potential abortive infection, i.e. parasites initiate development in a ‘wrong’ host, in which sporozoites initiate exo-erythrocytic development, which is then aborted, resulting in merozoites and gametocytes do not appear (Ciloglu et al. 2020b). Abortive infections are ‘dead ends’ of transmission, but might still be virulent (Valkiūnas and Iezhova 2017) and therefore are important to determine as such. Furthermore, microscopy can quantify infection intensity. Quantifying the parasitemia as average number of parasites in one affected host is important, as infection intensity appears to be a more reliable predictor of the parasite virulence than prevalence, given as the number of infected animals per total number of animals (Sol et al. 2003). The parasitemia was rather low for the majority of our samples, indicating mainly chronic instead of acute infections. Pathological effects are expected to be higher at the acute infection than at chronic stages (Townsend et al. 2018).

The applied nested PCR method may have underestimated mixed infections of *Haemoproteus* and *Plasmodium*. Therefore, we have used a second PCR assay. Ciloglu et al. (2019) stated that the multiplex PCR assay was designed for amplification of *H*. (*Parahaemoproteus*) and that it needs to be tested whether the amplification of primers works for *H*. (*Haemoproteus*). We could show that the primers amplify *H*. (*Haemoproteus*) infections, but display them at the same band height as *Plasmodium* infections. Hence, it was not possible to distinguish between *H*. (*Haemoproteus*) and *Plasmodium* infections according to obtained PCR bands. Therefore, the multiplex PCR assay seems not effective for species that are prone to *H*. (*Haemoproteus*) infections. Besides the order of Columbiformes, this mainly applies to the orders Pelecaniformes, Charadriiformes and Suliformes (Valkiūnas 2005; Levin et al. 2011, 2012; Merino et al. 2012). Occasionally, *H*. (*Haemoproteus*) also infects Passeriformes (Lacorte et al. 2013; Ferreira Junior et al. 2017). Our results indicate that the amplified fragment of non-coding mtDNA is more similar between *H*. (*Haemoproteus*) and *Plasmodium* than between *H*. (*Haemoproteus*) and *H*. (*Parahaemoproteus*). However, the PCR amplified a rather short fragment only. In general, there is no agreement on deep-level phylogenetic of avian haemosporidian parasites (Walther et al. 2016). While some authors refer to *H*. (*Haemoproteus*) and *H*. (*Parahaemoproteus*) as subgenera (Iezhova et al. 2011; Valkiūnas et al. 2013), others propose splitting them into two separate genera (Borner et al. 2016; Galen et al. 2018; Soares et al. 2020). Even though our study did not clarify the phylogenetic classification of *H*. (*Haemoproteus*) and *H*. (*Parahaemoproteus*), we could show that both infect columbiform birds. Several studies stated that all *Haemoproteus* parasites found in birds of the order Columbiformes belong to *H*. (*Haemoproteus*) (Boundenga et al. 2017). In fact, our phylogenetic analyses placed most lineages infecting columbiform birds into the *H*. (*Haemoproteus*) clade, but some also clustered within the *H*. *(Parahaemoproteus*) clade (STRTUR01 and STRTUR05). These results are in line with a few other studies, which isolated *H*. (*Parahaemoproteus*) from columbiform species (Križanauskienė et al. 2013; Boundenga et al. 2017).

In conclusion, this study contributes to our understanding of the haemosporidian parasite diversity circulating in free-living birds of the order Columbiformes. In addition to reporting novel lineages and novel host species, the data obtained here contribute to improve our knowledge on the taxonomic relationship of avian haemosporidians and offer reference information to monitor likely future changes in parasite ranges and diversity as a consequence of climate change, representing a potential relevant risk for declining turtle doves.

## Supplementary information

ESM 1 (DOCX 279 kb)

ESM 2 (DOCX 1593 kb)

ESM 3 (DOCX 15 kb)

## Data Availability

Sequences are deposited in GenBank under accession numbers MT888848-60.

## References

[CR1] Adamik P, Emmenegger T, Briedis M (2016). Barrier crossing in small avian migrants: individual tracking reveals prolonged nocturnal flights into the day as a common migratory strategy. Sci Rep.

[CR2] Akinpelu AI (2008). Prevalence and intensity of blood parasites in wild pigeons and doves (Family: Columbidae) from Sasha Forest Reserve, Ile-Ife, Nigeria. Asian J Anim Vet Adv.

[CR3] Applegate JE, Beaudoin RL (1970). Mechanism of spring relapse in avian malaria: effect of gonadotropin and corticosterone. J Wildl Dis.

[CR4] Atkinson CT, Van Riper C (1991) Pathogenicity and epizootiology of avian haematozoa: *Plasmodium*, *Leucocytozoon* and *Haemoproteus*. In: Loye JE, Zuk M (eds.) Bird-parasite interactions: ecology, evolution and behaviour. Oxford Ornithology Series, pp 19–48

[CR5] Bensch S, Waldenström J, Jonzen N, Westerdahl H, Hansson B, Sejberg D, Hasselquist D (2007). Temporal dynamics and diversity of avian malaria parasites in a single host species. J Anim Ecol.

[CR6] Bensch S, Hellgren O, Peréz-Tris J (2009). MalAvi: a public database of malaria parasites and related haemosporidians in avian hosts based on mitochondrial cytochrome b lineages. Mol Ecol Resour.

[CR7] Bernotienė R, Palinauskas V, Iezhova T, Murauskaitė D, Valkiūnas G (2016). Avian haemosporidian parasites (Haemosporida): a comparative analysis of different polymerase chain reaction assays in detection of mixed infections. Exp Parasitol.

[CR8] Borner J, Pick C, Thiede J, Kolawole OM (2016). Phylogeny of haemosporidian blood parasites revealed by a multi-gene approach. Mol Phylogenet Evol.

[CR9] Boundenga L, Perkins SL, Ollomo B, Rougeron V, Leroy EM, Renaud F, Prugnolle F (2017). Haemosporidian parasites of reptiles and birds from Gabon, Central Africa. J Parasitol.

[CR10] Bunbury N, Barton E, Jones CG, Greenwood AG, Tyler KM, Bell DJ (2007). Avian blood parasites in an endangered columbid: *Leucocytozoon marchouxi* in the Mauritian Pink Pigeon *Columba mayeri*. Parasitology.

[CR11] Calderon L, Campagna L, Wilke T (2016). Genomic evidence of demographic fluctuations and lack of genetic structure across flyways in a long distance migrant, the European turtle dove. BMC Evol Biol.

[CR12] Carlson J, Martínez-Gómez JE, Valkiūnas G, Loiseau C, Bell DA, Sehgal RN (2013). Diversity and phylogenetic relationships of hemosporidian parasites in birds of Socorro Island, México and their role in the re-introduction of the Socorro Dove (*Zenaida graysoni*). J Parasitol.

[CR13] Chagas CRF, de Oliveira Guimarães L, Monteiro EF (2016). Hemosporidian parasites of free-living birds in the São Paulo Zoo, Brazil. Parasitol Res.

[CR14] Chakarov N, Kampen H, Wiegmann A, Werner D, Bensch S (2020). Blood parasites in vectors reveal a united blackfly community in the upper canopy. Parasites Vectors.

[CR15] Ciloglu A, Ellis VA, Bernotienė R, Valkiūnas G, Bensch S (2019). A new one-step multiplex PCR assay for simultaneous detection and identification of avian haemosporidian parasites. Parasitol Res.

[CR16] Ciloglu A, Ellis VA, Duc M, Downing PA, Inci A, Bensch S (2020a) Evolution of vector transmitted parasites by host switching revealed through sequencing of *Haemoproteus* parasite mitochondrial genomes. Mol Phylogenet Evol 153:106947. 10.1016/j.ympev.2020.106947 10.1016/j.ympev.2020.106947 32866615

[CR17] Ciloglu A, Ergen AG, Inci A (2020). Prevalence and genetic diversity of avian haemosporidian parasites at an intersection point of bird migration routes: Sultan Marshes National Park, Turkey. Acta Trop.

[CR18] Clark P, Boardman W, Raidal S (2009). Atlas of clinical avian hematology.

[CR19] Clark NJ, Clegg SM, Lima MR (2014). A review of global diversity in avian haemosporidians (*Plasmodium* and *Haemoproteus*: Haemosporida): new insights from molecular data. Int J Parasitol.

[CR20] Clark NJ, Clegg SM, Klaassen M (2016). Migration strategy and pathogen risk: non-breeding distribution drives malaria prevalence in migratory waders. Oikos.

[CR21] Cornelius JM, Zylberberg M, Breuner CW, Gleiss AC, Hahn TP (2014). Assessing the role of reproduction and stress in the spring emergence of haematozoan parasites in birds. J Exp Biol.

[CR22] Cosgrove CL, Wood MJ, Day KP, Sheldon BC (2008). Seasonal variation in *Plasmodium* prevalence in a population of blue tits *Cyanistes caeruleus*. J Anim Ecol.

[CR23] Cramp S (1985) Handbook of the birds of Europe, the Middle East and North Africa. Volume IV, Terns to Woodpeckers. Oxford University Press, Oxford

[CR24] Darriba D, Taboada GL, Doallo R, Posada D (2012). jModelTest2: more models, new heuristics and parallel computing. Nat Methods.

[CR25] Drummond AJ, Rambaut A (2007). BEAST: Bayesian evolutionary analysis by sampling trees. BMC Evol Biol.

[CR26] Drummond AJ, Suchard MA, Xie D, Rambaut A (2012). Bayesian phylogenetics with BEAUti and the BEAST 1.7. Mol Biol Evol.

[CR27] Dubiec A, Podmokła E, Zagalska-Neubauer M, Drobniak SM, Arct A, Gustafsson L, Cichoń M (2016). Differential prevalence and diversity of haemosporidian parasites in two sympatric closely related non-migratory passerines. Parasitology.

[CR28] Dunn JC, Outlaw DC (2019). Flying into the future: avian haemosporidians and the advancement of understanding host–parasite systems. Parasitology.

[CR29] Dunn JC, Stockdale JE, Bradford EL (2017). High rates of infection by blood parasites during the nestling phase in UK Columbids with notes on ecological associations. Parasitology.

[CR30] Earle RA, Batianello SS, Bennet GF, Krecek RC (1993). Histopathology and morphology of the tissue stages of *Haemoproteus columbae* causing mortality in Columbiformes. Avian Pathol.

[CR31] Ellis VA, Huang X, Westerdahl H (2020). Explaining prevalence, diversity, and host specificity in a community of avian haemosporidian parasites. Oikos.

[CR32] Emmenegger T, Bauer S, Dimitrov D, Marin JO, Zehtindjiev P, Hahn S (2018). Host migration strategy and blood parasite infections of three sparrow species sympatrically breeding in Southeast Europe. Parasitol Res.

[CR33] Ferraguti M, Martínez-de la Puente J, Garcia-Longoria L, Soriguer R, Figuerola J, Marzal A (2019). From Africa to Europe: evidence of transmission of a tropical *Plasmodium* lineage in Spanish populations of house sparrows. Parasites Vectors.

[CR34] Ferreira Junior FC, Rodrigues RA, Ellis VA, Leite LO, Borges MAZ, Braga EM (2017). Habitat modification and seasonality influence avian haemosporidian parasite distributions in southeastern Brazil. PLoS ONE.

[CR35] Figuerola J, Green AJ (2000). Hematozoan parasites and migratory behaviour in waterfowl. Evol Ecol.

[CR36] Fisher I, Ashpole J, Scallan D, Carboneras C, Proud T (compilers) (2018) International Single Species Action Plan for the conservation of the European Turtle-dove *Streptopelia turtur* (2018 to 2028). European Commission Technical Report xxx-2018

[CR37] Foronda P, Valladares B, Rivera-Medina JA, Figueruelo E, Abreu N, Casanova JC (2004). Parasites of *Columba livia* (Aves: Columbiformes) in Tenerife (Canary Islands) and their role in the conservation biology of the Laurel pigeons. Parasite.

[CR38] Galen SC, Borner J, Martinsen ES, Schaer J, Austin CC, West CJ, Perkins SL (2018). The polyphyly of *Plasmodium*: comprehensive phylogenetic analyses of the malaria parasites (order Haemosporida) reveal widespread taxonomic conflict. R Soc Open sci.

[CR39] Gernhard T (2008). Yule process. J Theor Biol.

[CR40] Godfrey RD, Fedynich AM, Pence DB (1987). Quantification of hematozoa in blood smears. J Wildl Dis.

[CR41] Gu X, Fu YX, Li WH (1995). Maximum likelihood estimation of the heterogeneity of substitution rate among nucleotide sites. Mol Biol Evol.

[CR42] Gupta DK, Jahan N, Gupta N (2011). New records of *Haemoproteus* and *Plasmodium* (Sporozoa: Haemosporida) of rock pigeon (*Columba livia*) in India. J Parasit Dis.

[CR43] Haag-Wackernagel D, Moch H (2004). Health hazards posed by feral pigeons. J Infect.

[CR44] Hegemann A, Matson KD, Versteegh MA, Tieleman BI (2012). Wild skylarks seasonally modulate energy budgets but maintain energetically costly inflammatory immune responses throughout the annual cycle. PLoS One.

[CR45] Hellard E, Cumming GS, Caron A, Coe E, Peters JL (2016). Testing epidemiological functional groups as predictors of avian haemosporidia patterns in southern Africa. Ecosphere.

[CR46] Hellgren O, Waldenström J, Bensch S (2004). A new PCR assay for simultaneous studies of *Leucocytozoon*, *Plasmodium*, and *Haemoproteus* from avian blood. J Parasitol.

[CR47] Hellgren O, Waldenström J, Peréz-Tris J (2007). Detecting shifts of transmission areas in avian blood parasites - a phylogenetic approach. Mol Ecol.

[CR48] Hellgren O, Wood MJ, Waldenström J, Hasselquist D, Ottosson U, Stervander M, Bensch S (2013). Circannual variation in blood parasitism in a sub-Saharan migrant passerine bird, the garden warbler. J Evol Biol.

[CR49] Heym EC, Kampen H, Krone O, Schäfer M, Werner D (2019). Molecular detection of vector-borne pathogens from mosquitoes collected in two zoological gardens in Germany. Parasitol Res.

[CR50] Huang X, Huang D, Liang Y (2020). A new protocol for absolute quantification of haemosporidian parasites in raptors and comparison with current assays. Parasites Vectors.

[CR51] Iezhova TA, Dodge M, Sehgal RN, Smith TB, Valkiūnas G (2011). New avian *Haemoproteus* species (Haemosporida: Haemoproteidae) from African birds, with a critique of the use of host taxonomic information in hemoproteid classification. J Parasitol.

[CR52] Jenkins T, Thomas GH, Hellgren O, Owens IPF (2012). Migratory behaviour of birds affects their coevolutionary reationship with blood parasites. Evolution.

[CR53] Jovani R, Tella JL (2006). Parasite prevalence and sample size: misconceptions and solutions. Trends Parasitol.

[CR54] Klei TR, DeGiusti DL (1975). Seasonal occurrence of *Haemoproteus columbae* Kruse and its vector *Pseudolynchia canariensis* Bequaert. J Wildl Dis.

[CR55] Knowles SCL, Palinauskas V, Sheldon BC (2010). Chronic malaria infections increase family inequalities and reduce parental fitness: experimental evidence from a wild bird population. J Evol Biol.

[CR56] Križanauskienė A, Iezhova TA, Sehgal RNM, Carlson JS, Palinauskas V, Bensch S, Valkiūnas G (2013) Molecular characterization of *Haemoproteus sacharovi* (Haemosporida, Haemoproteidae), a common parasite of columbiform birds, with remarks on classification of haemoproteids of doves and pigeons. Zootaxa 3613:085–094. 10.11646/zootaxa.3616.1.7 24758794

[CR57] Lachish S, Knowles SCL, Alves R, Wood MJ, Sheldon BC (2011). Fitness effects of endemic malaria infections in a wild bird population: the importance of ecological structure. J Anim Ecol.

[CR58] Lacorte GA, Felix GMF, Pinheiro RRB (2013). Exploring the diversity and distribution of Neotropical avian malaria parasites - a molecular survey from Southeast Brazil. PLoS ONE.

[CR59] Lee KA, Martin LB, Hasselquist D, Ricklefs RE, Wikelski M (2006). Contrasting adaptive immune defenses and blood parasite prevalence in closely related Passer sparrows. Oecologia.

[CR60] Leigh JW, Bryant D (2015). PopART: full-feature software for haplotype network construction. Methods Ecol Evol.

[CR61] Levin II, Valkiūnas G, Santiago-Alarcon D (2011). Hippoboscid-transmitted *Haemoproteus* parasites (Haemosporida) infect Galapagos Pelecaniform birds: evidence from molecular and morphological studies, with a description of *Haemoproteus iwa*. Int J Parasitol.

[CR62] Levin II, Valkiūnas G, Iezhove TA, O’Brien SL, Parker PG (2012). Novel *Haemoproteus* species (Haemosporida: Haemoproteidae) from the swallow-tailed gull (Lariidae), with remarks on the host range of hippoboscid-transmitted avian hemoproteids. J Parasitol.

[CR63] MalAvi (2020) MalAvi: a database for avian haemosporidian parasites. https://130.235.244.92/Malavi/ Download of the ‘Hosts and Sites Table’, accessed 13.03.2020

[CR64] Martínez J, Martínez-de La Puente J, Herrero J (2009). A restriction site to differentiate *Plasmodium* and *Haemoproteus* infections in birds: on the inefficiency of general primers for detection of mixed infections. Parasitology.

[CR65] Marx M, Korner-Nievergelt F, Quillfeldt P (2016). Analysis of ring recoveries of European turtle doves *Streptopelia turtur* - flyways, timing of migration and origins of hunted birds. Acta Orn.

[CR66] Marzal A, De Lope F, Navarro C, Møller AP (2005). Malarial parasites decrease reproductive success: an experimental study in a passerine bird. Oecologia.

[CR67] Merino S, Moreno J, Sanz JJ, Arriero E (2000). Are avian blood parasites pathogenic in the wild? A medication experiment in blue tits (*Parus caeruleus*). Proc Royal Soc B.

[CR68] Merino S, Hennicke J, Martínez J (2012). Infection by *Haemoproteus* parasites in four species of frigatebirds and the description of a new species of *Haemoproteus* (Haemosporida: Haemoproteidae). J Parasitol.

[CR69] Møller AP, Erriyzøe J (1998). Host immune defence and migration in birds. Evol Ecol.

[CR70] PECBMS (2020) Population Trends of Common European Breeding Birds. https://pecbms.info/trends-and-indicators/species-trends/ accessed 23.09.2020

[CR71] Pulgarín-R PC, Gomez C, Bayly NJ (2018). Migratory birds as vehicles for parasite dispersal? Infection by avian haemosporidians over the year and throughout the range of a long-distance migrant. J Biogeogr.

[CR72] Quillfeldt P, Arriero E, Martínez J, Masello JF, Merino S (2011). Prevalence of blood parasites in seabirds- a review. Front Zool.

[CR73] R Core Team (2016) R: a language and environment for statistical computing. Version 3.2.4. Vienna: R Foundation for Statistical Computing. https://www.Rproject.org/

[CR74] Rambaut A (2007) FigTree. https://tree.bio.ed.ac.uk/software/figtree/

[CR75] Reidelbach J, Christl H (2002). A quantitative investigation into the temporal and spatial variations in the emergence of adult blackflies (Diptera: Simuliidae) from the Breitenbach, a small upland stream in Germany. Limnologica.

[CR76] Reinoso-Pérez MT, Canales-Delgadillo JC, Chapa-Vargas L, Riego-Ruiz L (2016). Haemosporidian parasite prevalence, parasitemia, and diversity in three resident bird species at a shrubland dominated landscape of the Mexican highland plateau. Parasites Vectors.

[CR77] Ricklefs RE, Fallon SM (2002). Diversification and host switching in avian malaria parasites. Proc Royal Soc B.

[CR78] Rintamäki P, Huhta E, Jokimäki J, Squires-Parsons D (1999). Leucocytozoonosis and Trypanosomiasis in Redstarts in Finland. J Wildl Dis.

[CR79] Rouffaer LO, Steensels M, Verlinden M, Vervaeke M, Boonyarittichaikij R, Martel A, Lambrecht B (2018). Usutu virus epizootic and Plasmodium coinfection in Eurasian Blackbirds (Turdus merula) in Flanders, Belgium. J Wildl Dis.

[CR80] Santiago-Alarcon D, Outlaw DC, Ricklefs RE, Parker PG (2010). Phylogenetic relationships of haemosporidian parasites in New World Columbiformes, with emphasis on the endemic Galapagos dove. Int J Parasitol.

[CR81] Scaglione FE, Pregel P, Cannizzo FT, Pérez-Rodríguez AD, Ferroglio E, Bollo E (2015). Prevalence of new and known species of haemoparasites in feral pigeons in northwest Italy. Malar J.

[CR82] Shurulinkov P, Ilieva M (2009). Spatial and temporal differences in the blood parasite fauna of passerine birds during the spring migration in Bulgaria. Parasitol Res.

[CR83] Soares L, Young EI, Ricklefs RE (2020). Haemosporidian parasites of resident and wintering migratory birds in The Bahamas. Parasitol Res.

[CR84] Sol D, Jovani R, Torres J (2000). Geographical variation in blood parasites in feral pigeons: the role of vectors. Ecography.

[CR85] Sol D, Jovani R, Torres J (2003). Parasite mediated mortality and host immune response explain age-related differences in blood parasitism in birds. Oecologia.

[CR86] Tavassoli M, Esmaeilnejad B, Malekifard F, Mardani K (2018) PCR-RFLP detection of *Haemoproteus spp*. (Haemosporida: Haemoproteidae) in pigeon blood samples from Iran. Bulg J Vet Med 21:429-435. 10.15547/bjvm.2014

[CR87] Townsend AK, Wheeler SS, Freund D, Sehgal RNM, Boyce WM (2018). Links between blood parasites, blood chemistry, and the survival of nestling American crows. Ecol Evol.

[CR88] Valkiūnas G (2005) Avian malaria parasites and other Haemosporidia. CRC Press

[CR89] Valkiūnas G, Iezhova TA (2017). Exo-erythrocytic development of avian malaria and related haemosporidian parasites. Malar J.

[CR90] Valkiūnas G, Bairlein F, Iezhova TA, Dolnik OV (2004). Factors affecting the relapse of *Haemoproteus belopolskyi* infections and the parasitaemia of *Trypanosoma spp*. in a naturally infected European songbird, the blackcap, *Sylvia atricapilla*. Parasitol Res.

[CR91] Valkiūnas G, Bensch S, Iezhova TA, Križanauskienė A, Hellgren O, Bolshakov CV (2006). Nested cytochrome b polymerase chain reaction diagnostics underestimate mixed infections of avian blood haemosporidian parasites: Microscopy is still essential. J Parasitol.

[CR92] Valkiūnas G, Iezhova TA, Loisseau C, Sehgal RNM (2009). Nested cythochrome b polymerase chain reaction diagnostics detect sporozoites of haemosporidian parasites in peripheral blood of naturally infected birds. J Parasitol.

[CR93] Valkiūnas G, Iezhova TA, Evans E, Carlson JS, Martínez-Gómez JE, Sehgal RNM (2013). Two new Haemoproteus species (Haemosporida: Haemoproteidae) from columbiform birds. J Parasitol.

[CR94] von Blotzheim UG, Bauer KM (1994). Handbuch Der Vögel Mitteleuropas, Band.

[CR95] Waldenström J, Bensch S, Kiboi S, Hasselquist D, Ottosson U (2002). Cross-species infection of blood parasites between resident and migratory songbirds in Africa. Mol Ecol.

[CR96] Walther EL, Carlson JS, Cornel A, Morris BK, Sehgal RNM (2016). First molecular study of prevalence and diversity of avian haemosporidia in a Central California songbird community. J Ornithol.

[CR97] Yanga S, Martínez-Goméz JE, Sehgal RNM, Escalante P, Camacho FC, Bell DA (2011). A preliminary survey for avian pathogens in Columbiformes birds on Socorro Island, Mexico. Pac Conserv Biol.

[CR98] Yoshimura A, Koketsu M, Bando H (2014). Phylogenetic comparisons of avian haemospridian parasites from resident and migratory birds in northern Japan. J Wildl Dis.

[CR99] Yule GU (1925). Yule process. Philos Trans R Soc Lond B Biol Sci.

[CR100] Zhang Z, Schwartz S, Wagner L, Miller W (2000). A greedy algorithm for aligning DNA sequences. J Comput Biol.

[CR101] Zwarts L, Bijlsma RG, van der Kamp J, Wymenga E (2009). Living on the edge: wetlands and birds in a changing Sahel. Chapter 32: European turtle dove *Streptopelia turtur*.

