# Supplementary material for: Prevalence and genetic diversity of avian haemosporidian parasites in wild bird species of the order Columbiformes
Source: Parasitol Res. 2021 Feb 1;120(4):1405–20. doi: 10.1007/s00436-021-07053-7 (PMC7940316; doi:10.1007/s00436-021-07053-7)
Supplement: Supplementary file 1 — (DOCX 279 kb) [file 436_2021_7053_MOESM1_ESM.docx]

Supplementary Material


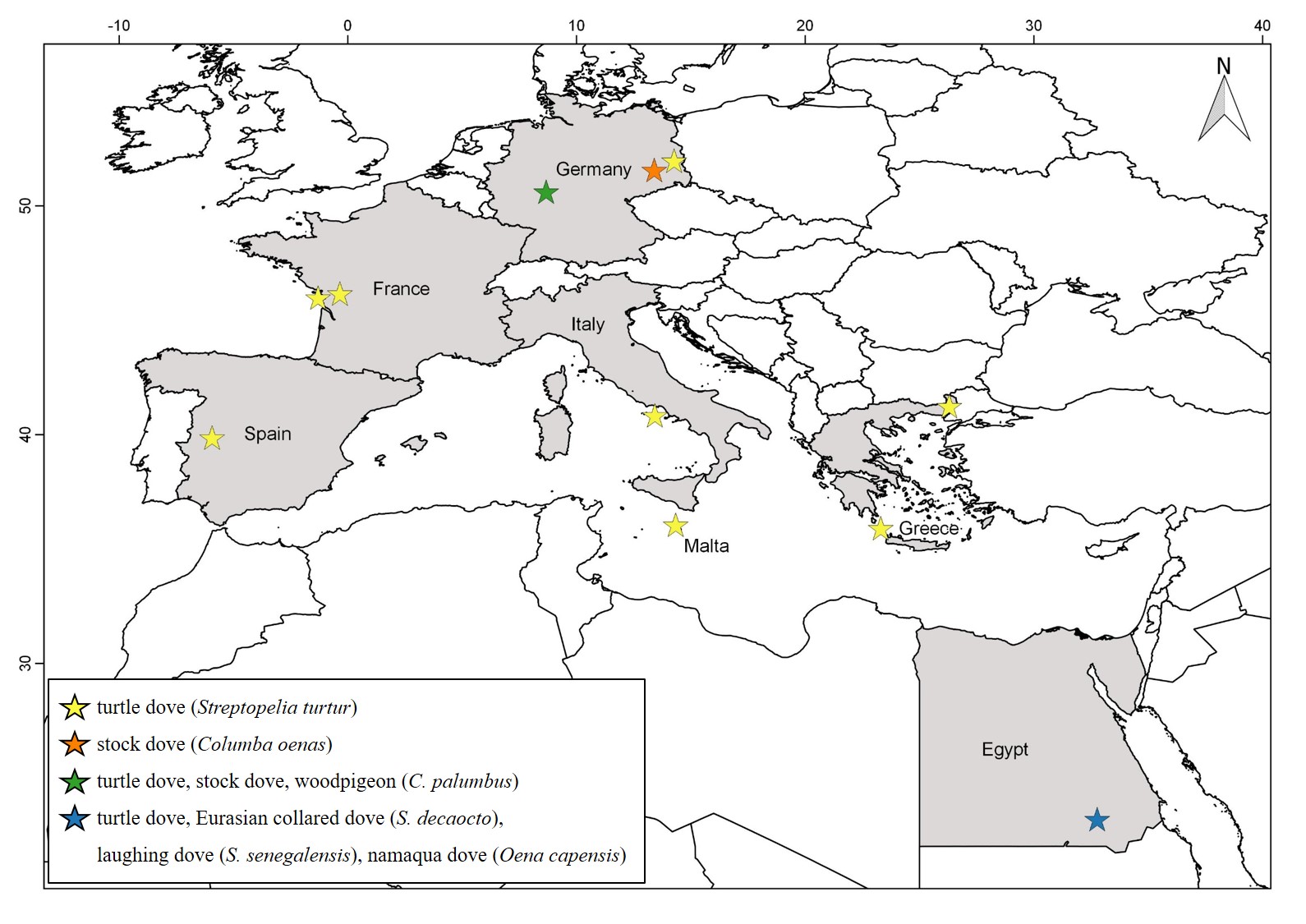


**Fig. S1** Map of sampling locations for blood samples of columbiform birds. The seven countries where sampling was conducted are shaded in grey. Sampling locations for each country are displayed by a star symbol. Sampled species are represented by the star colour.
