# Supplementary material for: Prevalence and genetic diversity of avian haemosporidian parasites in wild bird species of the order Columbiformes
Source: Parasitol Res. 2021 Feb 1;120(4):1405–20. doi: 10.1007/s00436-021-07053-7 (PMC7940316; doi:10.1007/s00436-021-07053-7)
Supplement: Supplementary file 2 — (DOCX 1593 kb) [file 436_2021_7053_MOESM2_ESM.docx]

Supplementary Material


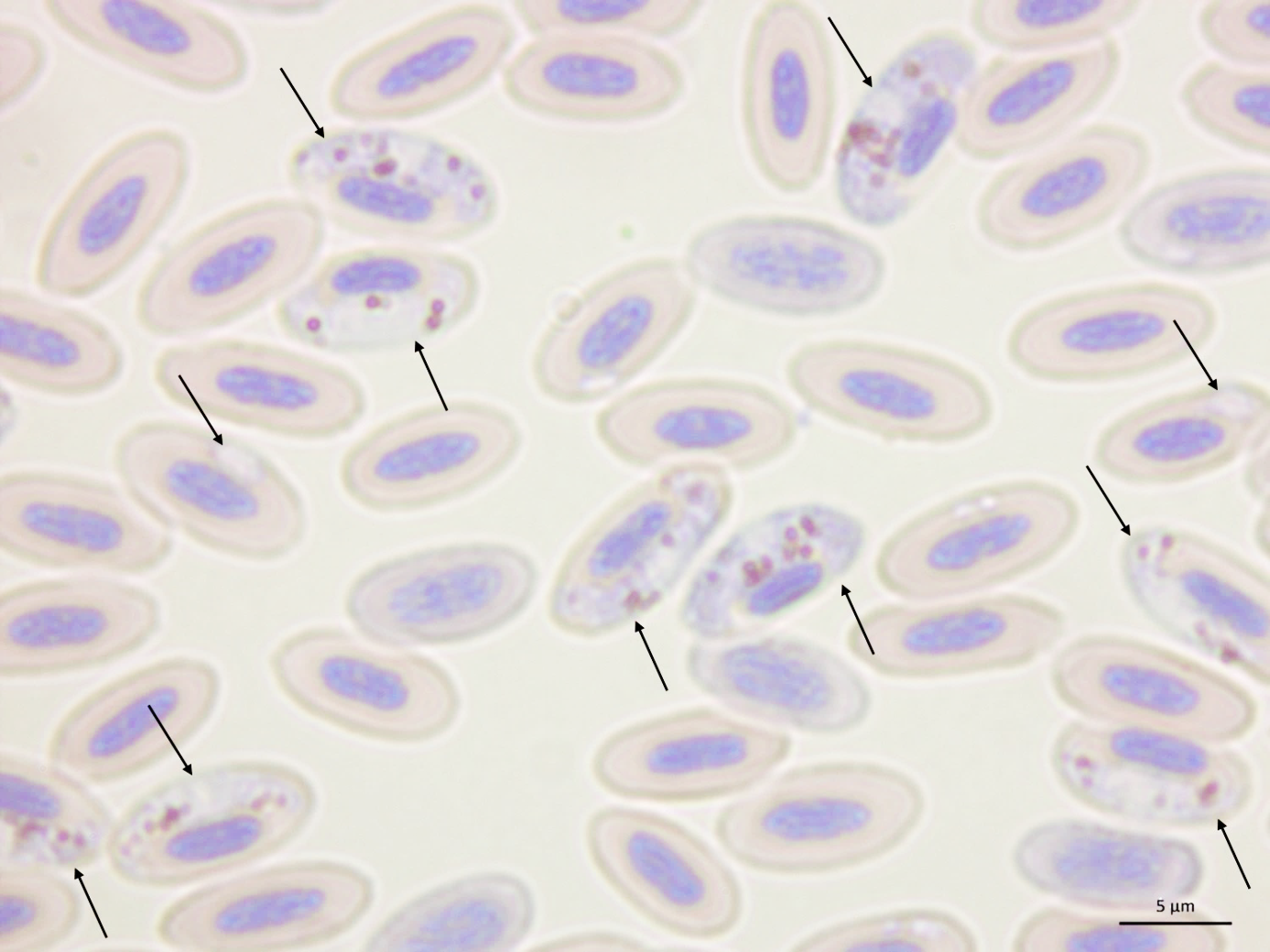


**Fig. S2** Giemsa-stained blood smear of an adult, male European turtle dove (*Streptopelia turtur*) sampled 2019 on Antikythira Island, Greece. Arrows show erythrocytes infected with *H*. (*Parahaemoproteus*) lineage STRTUR01 (light microscope PrimoStar and microscope camera Axio Cam ERc 5S, Carl Zeiss Microscopy GmbH).
