# Supplementary material for: Prevalence and genetic diversity of avian haemosporidian parasites in wild bird species of the order Columbiformes
Source: Parasitol Res. 2021 Feb 1;120(4):1405–20. doi: 10.1007/s00436-021-07053-7 (PMC7940316; doi:10.1007/s00436-021-07053-7)
Supplement: Supplementary file 3 — (DOCX 15 kb) [file 436_2021_7053_MOESM3_ESM.docx]

Supplementary Material

**Table S1** Lineage names and associated GenBank accession numbers for avian haemosporidian lineages (n = 109) used for phylogenetic tree construction based on a Bayesian analysis. Lineages found in the present study are shown in bold. Newly discovered lineages are marked with ‘*’.

| **Lineage name** | **GenBank accession no.** |  | **Lineage name** | **GenBank accession no.** |
| --- | --- | --- | --- | --- |
| **AEMO02** | MT888855 |  | COLMIN01 | MH457268 |
| AFR044 | KM056450 |  | COLPAL01 | KJ488710 |
| AFR070 | KM056461 |  | COLPAL02 | KJ488803 |
| AFR109 | KM056421 |  | **COLPAL03*** | MT888851 |
| AFR112 | KM056423 |  | **COLPAL04*** | MT888852 |
| **AFR119** | KM056425 |  | COLPAS04 | JN788935 |
| AFR120 | KM056426 |  | COLPAS06 | KU562226 |
| AFR178 | KM056496 |  | COLPLU02 | MH457387 |
| AFR220 | KM056535 |  | COLTAL01 | GU296214 |
| ALEMAD01 | MF442612 |  | COLTAL02 | MH457459 |
| BAFLA04 | JX029861 |  | COLTAL03 | MK695447 |
| CLAPRE01 | KU562242 |  | COPIC01 | KP686106 |
| COCOR03 | JX867112 |  | COQUI05 | KU131585 |
| COCOR09 | AB741497 |  | COSQU01 | JX029921 |
| COCOR11 | KJ128988 |  | COSQU02 | KP686107 |
| COCOR12 | KJ128989 |  | COSQU03 | KP686098 |
| COCOR13 | KJ128991 |  | COSQU04 | KP686099 |
| COCOR15 | LC230130 |  | CYCYA01 | DQ241529 |
| COLBUC01 | GU296227 |  | ELACHI01 | MH457257 |
| COLIV03 | MN065191 |  | ESTMEL03 | JN661938 |
| **COLIV04** | MT888857 |  | FOUMAD02 | JN661926 |
| COLIV05 | AB741507 |  | GALLUS02 | AB250415 |
| COLIV06 | KU057964 |  | GEOFRE01 | MH457361 |
| COLIV07 | KY653761 |  | GEOMON01 | KU562207 |

| **Lineage name** | **GenBank accession no.** |  | **Lineage name** | **GenBank accession no.** |
| --- | --- | --- | --- | --- |
| GEOTRY01 | KT373865 |  | **STRTUR01** | MT888860 |
| **GRW02** | MT888853 |  | **STRTUR02** | KJ488786 |
| GRW06 | DQ368381 |  | **STRTUR03** | KJ488826 |
| HAECOL1 | AF495554 |  | **STRTUR04*** | MT888848 |
| LAMPUR03 | EU810655 |  | **STRTUR05*** | MT888849 |
| LEPPLU01 | GU296213 |  | SYAT05 | DQ847271 |
| LEPRUF02 | JX029909 |  | THACAE01 | KT373877 |
| LEPVER03 | MH457277 |  | TURABY01 | MG018657 |
| MACAMB01 | AY714193 |  | TURABY02 | MG018659 |
| METMEL01 | MK947509 |  | TURAFE03 | KX148095 |
| METMEL03 | MK947510 |  | UPUPA02 | KJ488831 |
| METMEL04 | MK947511 |  | VIGIL09 | MF817757 |
| METMEL05 | MK947512 |  | ZEAUR01 | MK695426 |
| **MILANS06** | MT888859 |  | ZEAUR02 | MK695474 |
| MITOM01 | KX171625 |  | ZEAUR03 | MK695475 |
| NESPIC01 | MF442604 |  | ZEAUR05 | GQ141564 |
| NYCNYC01 | KU057967 |  | ZEAUR07 | KY653812 |
| **OENCAP01*** | MT888850 |  | ZEGAL05 | GU296215 |
| PTIRIV01 | AY714139 |  | ZEGAL08 | JF833051 |
| PTISUP01 | AY714136 |  | ZEMAC01 | AY099032 |
| PTISUP02 | AY714137 |  | ZEMAC02 | JN788932 |
| **RTSR1** | KJ488785 |  | ZEMAC03 | JN788933 |
| SGS1 | AF495571 |  | ZEMAC05 | JN788936 |
| SPISEN01 | MF374494 |  | ZEMAC11 | JN788943 |
| SPISEN02 | MF374495 |  | ZEMAC12 | JN788944 |
| STPIC01 | JN032657 |  | ZEMAC13 | JN788945 |
| STRORI01 | AB741491 |  | ZEMAC14 | JN788947 |
| **STRORI02** | MT888858 |  | ZEMAC15 | JN788948 |
| STRORI03 | AB741511 |  | ZEMAC16 | JN788950 |
| STRORI04 | LC230148 |  | ZEMAC17 | KY653811 |
| STRORI05 | LC230152 |  |  |  |
